# Polarity Specific Suppression Effects of Transcranial Direct Current Stimulation for Tinnitus

**DOI:** 10.1155/2014/930860

**Published:** 2014-04-10

**Authors:** Kathleen Joos, Dirk De Ridder, Paul Van de Heyning, Sven Vanneste

**Affiliations:** ^1^Department of Neurosurgery, University Hospital Antwerp, 2650 Edegem, Belgium; ^2^Department of Translational Neuroscience, Faculty of Medicine, University of Antwerp, 2650 Edegem, Belgium; ^3^Department of Surgical Sciences, Dunedin School of Medicine, University of Otago, Dunedin 9054, New Zealand; ^4^BRAI^2^N & TRI, Sint Augustinus Hospital, 2610 Wilrijk, Belgium; ^5^Department of Otorhinolaryngology and Head & Neck Surgery, University Hospital Antwerp, 2650 Edegem, Belgium; ^6^School of Behavioral and Brain Sciences, The University of Texas at Dallas, TX 75235, USA

## Abstract

Tinnitus is the perception of a sound in the absence of an external auditory stimulus and affects 10–15% of the Western population. Previous studies have demonstrated the therapeutic effect of anodal transcranial direct current stimulation (tDCS) over the left auditory cortex on tinnitus loudness, but the effect of this presumed excitatory stimulation contradicts with the underlying pathophysiological model of tinnitus. Therefore, we included 175 patients with chronic tinnitus to study polarity specific effects of a single tDCS session over the auditory cortex (39 anodal, 136 cathodal). To assess the effect of treatment, we used the numeric rating scale for tinnitus loudness and annoyance. Statistical analysis demonstrated a significant main effect for tinnitus loudness and annoyance, but for tinnitus annoyance anodal stimulation has a significantly more pronounced effect than cathodal stimulation. We hypothesize that the suppressive effect of tDCS on tinnitus loudness may be attributed to a disrupting effect of ongoing neural hyperactivity, independent of the inhibitory or excitatory effects and that the reduction of annoyance may be induced by influencing adjacent or functionally connected brain areas involved in the tinnitus related distress network. Further research is required to explain why only anodal stimulation has a suppressive effect on tinnitus annoyance.

## 1. Introduction

Tinnitus is a common perception of a tone, hissing, or buzzing sound in the absence of an external auditory stimulus and is therefore also described as a phantom sound [1]. It is continuously present in about 10–15% of the Western population [2], of which 2–4% perceives severe interference with their quality of life [3] as it is often associated with symptoms such as annoyance [4], anxiety [5], depression [5], and sleep disturbances [6]. In elderly patients, the prevalence percentages can even rise up to 33% [7, 8], most likely due to the higher prevalence of hearing impairment [9].

Although no consensus has been reached about the neurophysiological model of tinnitus, it is proposed that tinnitus is related to either auditory deafferentation [10–15] or a deficit in noise cancelling [16, 17] or a combination of both [18]. Moreover, tinnitus has been hypothesized to be the expression of a thalamocortical dysrhythmia, in which there is a constant (pathologic) coupled theta-gamma band activity (theta: 4–7 Hz, gamma > 30 Hz) due to hyperpolarization of specific thalamic nuclei [19]. In normal circumstances, incoming auditory stimuli induce a transient gamma band activity [20] in a restricted area [21], which binds by nesting on theta activity [22, 23], that is, a transient coupling between a high- and low-frequency band of ongoing electrical activity in the human brain [22]. This is mediated by a shift of alpha activity towards high-frequency gamma band oscillations [20]. In a deafferented state, neural activity is shifted towards theta band activity [24] which in turn leads to a decreased lateral inhibition mediated by *γ*-amino butyric acid [25] and results in a persistent and thus pathological gamma activity of the neighboring neurons, also known as the “edge effect” [19, 25]. This sustained gamma band activity present in temporal areas is related to tinnitus as observed by quantitative electroencephalography (qEEG) [26] and magnetoelectroencephalography (MEG) [12, 19, 25]. The coupled presence of theta and gamma activity in tinnitus has also been demonstrated by recordings from an implanted electrode overlying the auditory cortex in a tinnitus patient [27]. Furthermore, this theta-gamma coupled activity is maximal at the area of blood oxygenation level dependent (BOLD) activation evoked by tinnitus-matched sound presentation in the magnetic resonance imaging (MRI) scanner [27], suggesting that the BOLD signal can demonstrate pathological tinnitus related activity in the auditory cortex [28]. Moreover, a positive correlation has been demonstrated between the amount of gamma band activity in the auditory cortex and the perceived tinnitus loudness in the contralateral auditory cortex [29].

The involvement of the auditory cortex cannot only be concluded from neuroimaging and electrophysiological measurements but it can also be claimed from the results of both invasive and noninvasive neuromodulation studies. Extradural stimulation of the secondary auditory cortex by a fMRI-guided neuronavigated electrode implant, based on BOLD activation evoked by tinnitus-matched sound [30–32], could partially or completely suppress tinnitus in 67% of the patients who perceived a tinnitus suppressive effect by transcranial magnetic stimulation (TMS) [33]. Several studies explored the therapeutic effect of TMS over the temporoparietal cortex in the treatment of tinnitus demonstrating that repetitive high frequency TMS, that is, increasing cortical excitability, causes a tinnitus suppression effect in about 50% of the patients [34–37]. Interestingly, both single [38, 39] and repetitive sessions [40–44] of low-frequency TMS over the temporoparietal cortex have been successful in the treatment of tinnitus as well. Another noninvasive neuromodulation technique applied in the treatment of tinnitus is transcranial direct current stimulation (tDCS). tDCS is applied by two surface electrodes, one anode and one cathode, of which one or two are placed over the scalp. Although a part of the applied current will be shunted by scalp tissue, a substantial part reaches the brain [45]. It has been demonstrated that anodal direct current stimulation induces depolarization of the underlying neurons, while cathodal stimulation leads to hyperpolarization [46], mainly by influencing the resting membrane potential. Combining this with the above-mentioned alterations in neural activity observed in tinnitus patients leads to the suggestion that the cathode overlying the auditory cortex should exert a tinnitus suppressing effect while the anode should have a potentially tinnitus worsening effect. However, Fregni et al. could obtain a transient suppression on tinnitus loudness using anodal tDCS over the left temporoparietal cortex [36]. These results were replicated by Garin et al. [47], albeit different stimulation parameters were used. It should be noticed that these tDCS results are rather paradoxical to the previously proposed model of tinnitus, whereas tinnitus is related to neural hyperactivity of the auditory cortex. Therefore, we retrospectively looked at our data in 175 tinnitus patients in which the effects of a single session of anodal or cathodal tDCS over the auditory cortex was evaluated both for tinnitus loudness and annoyance.

## 2. Methods

### 2.1. Subjects

175 patients (116 male, 59 female) with chronic tinnitus (>1 year) received auditory cortex tDCS (Table 1). The mean age of the patients was 48.46 years (Sd = 13.27) and the mean tinnitus duration was 5.56 years (Sd = 6.82). All patients underwent a single session of tDCS performed in the treatment of tinnitus at the Tinnitus Research Initiative (TRI), Antwerp. Of these 175 patients, 43 received tDCS with an intensity of 1.5 mA, while 132 patients received tDCS of 2.0 mA. The applied stimulation intensity and the side of stimulation were chosen randomly. Individuals with pulsatile tinnitus, Ménière disease, otosclerosis, chronic headache, and neurological disorders such as brain tumors and individuals being treated for mental disorders were not included in the study in order to obtain a homogeneous sample. Therefore, all patients included for this study firstly underwent a complete audiological, ENT, and neurological investigation. In addition, several technical investigations were performed including MRI of the brain. Collection of the data was under approval of IRB UZA OGA85. All patients gave an informed consent.

### 2.2. Transcranial Direct Current Stimulation

For the application of tDCS, a pair of electrodes with a surface of 35 cm² was placed in saline-soaked sponges. Both electrodes, one anode and one cathode, were connected to a battery, which delivers a constant current with a maximum output of 10 mA (Neuroconn; http://www.neuroconn.de/). The stimulation was applied for 20 minutes with a current intensity of 1.5 or 2.0 mA in a quiet room. These stimulation parameters are considered to be safe and without any significant side effects [48, 49]. The anodal or cathodal electrode was placed over the left or right auditory cortex, that is, T3 or T4 of the International 10/20 Electroencephalogram System, respectively, while the reference electrode was placed on the contralateral arm. An advantage of placing the reference electrode extracephalic is that interference from the reference electrode can be avoided [50], contrary to most previous studies in which they made use of a bicephalic electrode positioning.

### 2.3. Evaluation

A numeric rating scale (NRS) for tinnitus loudness (“How loud do you perceive your tinnitus? 0: no tinnitus and 10: as loud as imaginable”) and annoyance (“How annoying is your tinnitus? 0: not annoying and 10: extremely annoying”) was asked before and directly after tDCS stimulation.

### 2.4. Statistical Analysis

Calculations were performed using SPSS 22 software package. A repeated measure ANOVA was conducted with tinnitus loudness pre- and posttreatment as within-subjects variable and stimulation parameter (cathodal versus anodal stimulation) and location (left versus right auditory cortex) as between-subjects variables for the group receiving 1.5 mA tDCS. Likewise, a repeated measure ANOVA was performed with tinnitus annoyance pre- and posttreatment as within-subjects variable and stimulation parameter (cathodal versus anodal stimulation) and location (left versus right auditory cortex) as between-subjects variables for the group receiving 1.5 mA tDCS. Both analyses were repeated for the group of patients receiving 2.0 mA tDCS.

To further interpret the interaction effect, we conducted a simple contrast analysis. This latter method has the advantage that a specific contrast can be compared within the full model, without separating the groups (stimulation, location) into different independent statistical tests excluding part of the variance. Although the repeated measures ANOVA was conducted as a whole model (including the different main effects as well as the interaction effects), we report the results in different subheadings for reasons of clarity.

In addition, a post hoc analysis was performed for the 2.0 mA group to control for tinnitus lateralization as only in this group significant results could be obtained. Therefore, a repeated measures ANOVA was performed with the pre-and posttreatment as within-subjects variable and stimulation (anodal versus cathodal auditory cortex stimulation) as between-subjects variable for both loudness and annoyance while we controlled for tinnitus lateralization (left-sided, right-sided, or bilateral tinnitus). This is necessary as it could be assumed that the effect of treatment is influenced by tinnitus laterality and therefore, depending on the side of the tinnitus, stimulation should be applied over a specific side or with a specific polarity to gain a therapeutic effect.

## 3. Results

### 3.1. ****1.5 mA tDCS

**Figure 1:**
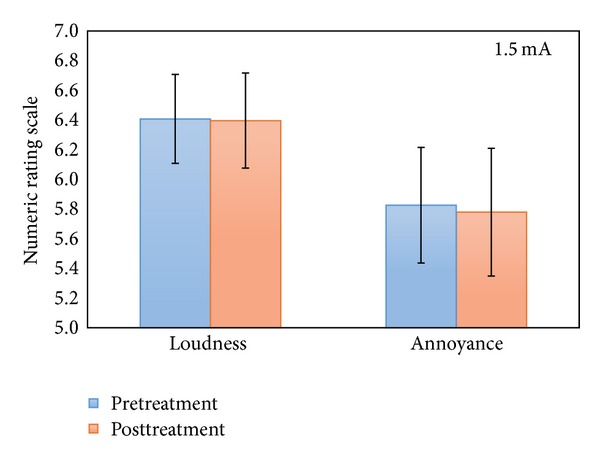
NRS loudness and annoyance pre- and posttreatment for the 1.5 mA group.

A repeated measures ANOVA with the pre- and posttreatment as within-subjects variable and stimulation (anodal versus cathodal auditory cortex stimulation) and location (left versus right auditory cortex) as between-subjects variables for both loudness and annoyance was performed for the patients receiving tDCS with an intensity of 1.5 mA. This analysis revealed no significant effect for tinnitus loudness or annoyance (see Figure 1) and no significant interaction effect could be demonstrated with polarity. Moreover, no additional main or interaction effects could be demonstrated for tinnitus loudness or annoyance in the patients receiving 1.5 mA. In addition, when defining tDCS responders as those patients having a difference in NRS loudness or annoyance greater than zero when comparing pre- to poststimulation NRS scores, only 3 out of 43 patients experienced a suppressive effect of tDCS on tinnitus loudness, while 2 patients experienced a suppressive effect on tinnitus related annoyance. Responders were only present in the group of patients receiving cathodal stimulation.

### 3.2. ****2.0 mA tDCS 

#### 3.2.1. Pre- versus Posttreatment

**Figure 2:**
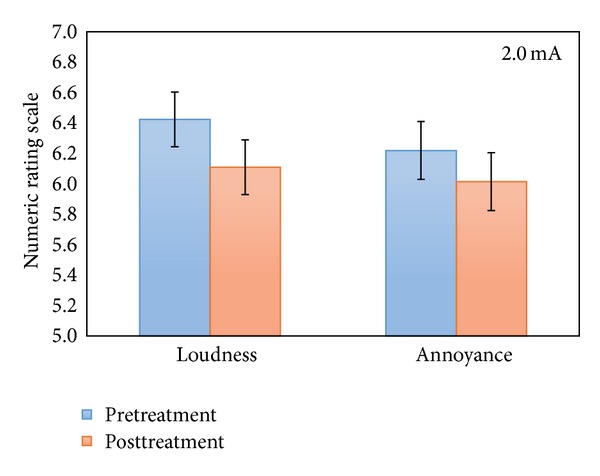
NRS loudness and annoyance pre- and posttreatment for the 2.0 mA group.

A repeated measures ANOVA with the pre- and posttreatment as within-subjects variable and stimulation (anodal versus cathodal auditory cortex stimulation) and location (left versus right auditory cortex) as between-subjects variables for both loudness and annoyance was performed for the patients receiving tDCS with an intensity of 2.0 mA. This analysis yielded a significant treatment effect for tinnitus loudness (*F*(1,130) = 15.90, *P* < .001) indicating that after the treatment session (M = 6.11, Sd = 1.78) tinnitus patients had a decrease of their tinnitus loudness in comparison to pretreatment (M = 6.42, Sd = 1.76) (see Figure 2), although only 23 patients out of 132 experienced a tinnitus suppressing effect. Of these responders, 10 received anodal stimulation, while 13 received cathodal tDCS. In addition, a significant decrease (*F*(1,130) = 13.79, *P* < .001) of tinnitus annoyance was observed when posttreatment scores (M = 6.02, Sd = 1.89) were compared to pretreatment scores (M = 6.22, Sd = 1.82) (see Figure 2), while only 15 patients perceived a reduction of tinnitus related annoyance. 9 Of these patients received anodal tDCS and 6 were given cathodal stimulation.

#### 3.2.2. Pre- versus Posttreatment Dependence on Anodal or Cathodal Stimulation

**Figure 3:**
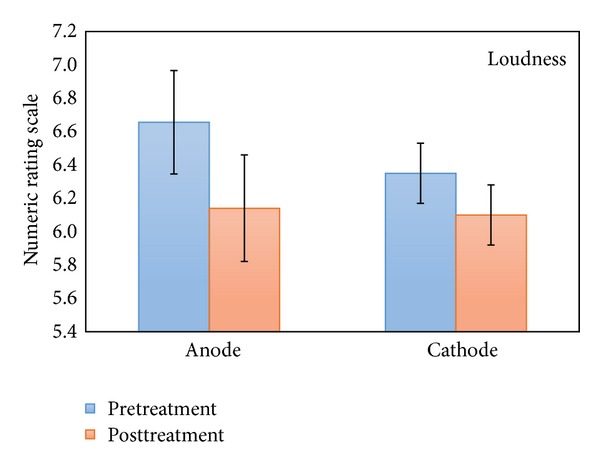
NRS loudness pre- and posttreatment (anodal and cathodal) for the 2.0 mA group.

**Figure 4:**
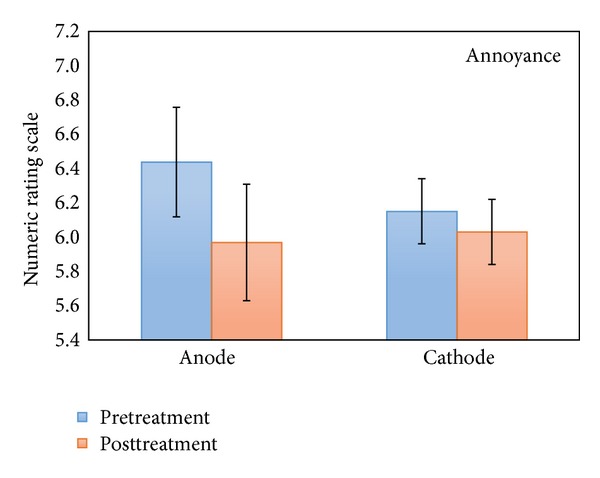
NRS annoyance pre- and posttreatment (anodal and cathodal) for the 2.0 mA group.

This repeated measures ANOVA with pre- and posttreatment as within-subjects variable and stimulation (anodal versus cathodal auditory cortex stimulation) and location (left versus right auditory cortex) as between-subjects variable demonstrated no significant interaction effect between pre- and posttreatment for loudness and stimulation polarity (see Figure 3), although a significant interaction effect between polarity and pre- and posttreatment measurement was observed for tinnitus annoyance (*F*(1,130) = 3.98, *P* < .05). A simple contrast analysis revealed that for tinnitus annoyance there was a significant effect for anodal stimulation when comparing prestimulation (M = 6.44, Sd = 1.58) to poststimulation (M = 5.97, Sd = 1.69)  (*F*(1,130) = 10.56, *P* = .001), but no significant effect was obtained for cathodal stimulation between pre- (M = 6.15,Sd = 1.89) and poststimulation (M = 6.03, Sd = 1.95) (see Figure 4).

#### 3.2.3. Other Main and Interaction Effects

Our repeated measures ANOVA revealed no significant effect for the two-way interaction between treatment (pre versus post) and location (left versus right auditory cortex) or for the three-way interaction between treatment (pre versus post), stimulation (anodal versus cathodal), and location (left versus right auditory cortex) for tinnitus loudness or annoyance.

In addition, no significant effects were demonstrated for loudness or annoyance for the between-subjects variables stimulation (anodal versus cathodal) and location (left versus right auditory cortex). Furthermore, no significant effect was obtained for the two-way interaction between stimulation (anodal versus cathodal) and location (left versus right auditory cortex) for tinnitus loudness or annoyance.

#### 3.2.4. Controlling for Tinnitus Lateralization

A repeated measures ANOVA with pre- and posttreatment as within-subjects variable and stimulation (anodal versus cathodal stimulation) as between-subjects variable for loudness was performed, while we controlled for tinnitus lateralization (left-sided, right-sided, or bilateral tinnitus). This analysis revealed no significant effect for tinnitus lateralization or an interaction effect between treatment (pre versus post) and tinnitus lateralization. Moreover, no significant interaction effect could be demonstrated between pre- and posttreatment and stimulation (anodal versus cathodal).

The same analysis was performed for tinnitus related annoyance, demonstrating that there was no significant effect for tinnitus lateralization or interaction effect between treatment (pre versus post) and tinnitus lateralization. However, a significant interaction effect between pre- and posttreatment and stimulation (anodal versus cathodal) for the 2 mA condition remained (*F*(1,130) = 4.11, *P* < 0.05).

## 4. Discussion 

Our results demonstrate an overall significant suppressive effect of tDCS applied over the auditory cortex for tinnitus loudness and annoyance, but only when tDCS was applied with an intensity of 2 mA. This overall effect on tinnitus annoyance was however likely mediated by the specific polarity of stimulation. That is, a significantly more pronounced effect was demonstrated for anodal than for cathodal stimulation when the electrode was placed over the auditory cortex, irrespective of whether stimulation was applied over the left or right auditory cortex.

Currently, only limited studies have been performed in which single session tDCS has been applied over the auditory cortex. Both Fregni et al. [36] and Garin et al. [47] could obtain a significant reduction of tinnitus loudness when anodal stimulation was applied over the left temporoparietal area, but not when the cathode was placed over the left temporoparietal cortex. However, it has to be mentioned that 6 out of 20 patients reported a reduction of their tinnitus loudness with cathodal stimulation, even though results were not statistically significant in the experiment of Garin et al. In addition to the positive effects on tinnitus loudness, neither anodal nor cathodal stimulation could induce a significant reduction on tinnitus discomfort [47]. The most plausible explanation for the different results compared to our study, that is, a significant reduction of tinnitus loudness independent of polarity and a suppressive effect on annoyance mainly mediated by anodal stimulation, is the difference in stimulation parameters. Both Fregni and Garin used tDCS with an intensity of only 1 mA and this with duration of 3 minutes in the study of Fregni, while the study of Garin as well as our own applied tDCS for 20 minutes. Based on these observations and our negative results for both tinnitus loudness and annoyance when tDCS was applied with an intensity of 1.5 mA, we may suggest that tDCS intensity is a decisive parameter and that cathodal stimulation might require a higher stimulation intensity to gain equally pronounced effects as anodal stimulation. Recently, Shekhawat et al. performed anodal stimulation over the left temporoparietal cortex and revealed that 2.0 mA was the more effective stimulation parameter when compared to an intensity of 1.0 mA [51].

Although the effects of tDCS on tinnitus loudness are more pronounced when a higher stimulation intensity is used, the effects of tDCS on tinnitus loudness are not influenced by polarity or by the side of stimulation. If we look at the pathophysiological model of tinnitus, one of the most consistent findings is the constant presence of pathological gamma activity in the auditory cortex demonstrated with both MEG [12, 19, 25] and qEEG [26], as well as on implanted electrodes [27]. Moreover, a strong positive correlation has been found between gamma oscillations in the contralateral auditory cortex and tinnitus intensity [29]. Because cathodal stimulation has been shown to have an inhibitory effect, it seems plausible that cathodal stimulation should induce the most pronounced effect as it can counteract this pathologic hyperactivity, but based on our results and the results of the above mentioned studies, other mechanisms should be considered. One possible explanation is that both cathodal and anodal stimulation have a disrupting effect on ongoing network activity, independent of their inhibitory or excitatory effect. Moreover, anodal stimulation may decrease pathologic hyperactivity of surrounding brain areas, by either competitive or inhibitory effects [36]. An interesting remark we need to make is that, in a recent study, in which cathodal tDCS of 1.0 and 2.0 mA was applied over the motor cortex in healthy subjects, reversed effects were obtained. More precisely, application of 2.0 mA cathodal tDCS resulted in cortical excitability enhancement instead of inhibition, similar to the results obtained with 2.0 mA anodal tDCS [52]. They suggest that the reversed effects are possibly due to the dependency of the direction of plasticity from the amount of neuronal calcium influx caused by the stimulation or that the resulting neuronal excitability change is determined by the axonal orientation relative to the electric field vector.

Besides the suppressive effect of tDCS on tinnitus loudness, a decrease of tinnitus related annoyance could be identified. Moreover, a significant interaction effect could be demonstrated between overall treatment and stimulation polarity with further analysis revealing that only anodal stimulation has a significant effect on annoyance. The amount of annoyance correlates with an alpha network consisting of the amygdala-anterior cingulate cortex-insula-parahippocampus-dorsolateral prefrontal cortex (DLPFC) [53, 54] and annoyance in tinnitus patients is related to the alpha and beta activity in the dorsal anterior cingulate cortex [53, 54]. Performing single-session, bilateral tDCS with the anode placed over the right DLPFC and the cathode over the left DLPFC induces a suppressive effect on annoyance [55, 56]. Similarly, repeated sessions of bifrontal tDCS could induce a small clinical effect on tinnitus discomfort [57]. Interesting results as the DLPFC has been shown to be involved in depression [58, 59], anxiety, and the affective component of pain [60], as well as in the processing of aversive auditory stimuli [61] and the sensory and emotional aspects of tinnitus [10, 53]. Most likely, our stimulation design does not only influence the underlying auditory cortex but also adjacent and functionally connected brain regions, for example the DLPFC. As we made use of an extracephalic reference electrode, the applied electric current will show a more widespread distribution than when bicephalic electrode positions are used [62]. This might as well explain why Garin et al. [47] could not find a significant effect on tinnitus discomfort, besides the lower current intensities used, as they positioned the reference electrode on the right scalp. But although we suggest that tDCS targeting the auditory cortex may influence the tinnitus related distress network, we cannot yet explain why only anodal stimulation, and this independently of the side of stimulation, leads to a decrease in annoyance. However, we should notice that recently a correlation between tinnitus distress and grey matter volume in bilateral auditory areas using voxel based morphometry (VBM) was identified [63], suggesting that stimulation of the auditory cortex may have a direct influence on tinnitus related annoyance as well, analogous to what has been seen in implants on the auditory cortex for tinnitus suppression [31, 33].

Some limitations of this study should be noted. Firstly, although we included 175 patients, only 43 patients received tDCS with an intensity of 1.5 mA and of the remaining 132 patients with 2.0 mA tDCS, only 32 patients received anodal stimulation while 100 patients received cathodal stimulation. The reason for this unequal distribution is that this was a retrospective study. Furthermore, it was not a placebo controlled study, but it has been previously demonstrated that sham stimulation applied over the left temporal lobe does not induce a significant effect [36] and the observation that no significant results could be obtained with 1.5 mA supports the fact that our results are not likely to be due to a placebo effect. Moreover, the main scope of our study was to explore the different effects of anodal and cathodal stimulation, rather than the therapeutic effect of tDCS in tinnitus per se.

In conclusion, we observed an overall suppressive effect for tDCS applied over the auditory cortex on tinnitus loudness and annoyance when performed with an intensity of 2.0 mA, but in contrast to previous tDCS studies the effect on tinnitus loudness was independent of polarity. For tinnitus annoyance on the other hand, a significant influence of stimulation polarity could be demonstrated, with a more pronounced effect for anodal than cathodal stimulation. Based on these observations, we suggest that reduction of tinnitus intensity may be caused by a disrupting effect on ongoing hyperactivity in the auditory cortex and functionally related brain areas, independent of polarity. Moreover, we hypothesize that auditory cortex stimulation may influence the tinnitus related distress network, but further research has to be performed to reveal why only anodal stimulation, independent of the side of stimulation, is capable of reducing annoyance.

## Figures and Tables

**Table 1:** Patient characteristics and stimulation parameters.

	Stimulation intensity	
	1.5 mA	2.0 mA
Age (years)	48.37 ± 15.73	48.49 ± 12.44
Gender (female/male)	19/24	40/92
Tinnitus laterality (left/right/bilateral)	17/4/22	29/19/84
NRS tinnitus loudness	6.41 ± 1.37	6.42 ± 1.76
NRS tinnitus annoyance	5.83 ± 1.69	6.22 ± 1.82

Stimulation (anodal/cathodal)	7/36	32/100
Anodal stimulation: location (left/right)	2/5	16/16
Cathodal stimulation: location (left/right)	22/14	44/56
